# The Core Techniques of Morenian Psychodrama: A Systematic Review of Literature

**DOI:** 10.3389/fpsyg.2018.01263

**Published:** 2018-07-24

**Authors:** Ana Cruz, Célia M. D. Sales, Paula Alves, Gabriela Moita

**Affiliations:** ^1^Psychiatry and Mental Health, Centro Hospitalar do Porto, Porto, Portugal; ^2^Center for Psychology at the University of Porto, Faculdade de Psicologia e de Ciências da Educação, Universidade do Porto, Porto, Portugal; ^3^Research Department of Primary Care and Population Health, University College London, London, United Kingdom; ^4^Institute of Psychiatry, Psychology and Neuroscience, King's College London, London, United Kingdom; ^5^Centro Lusíada de Investigação em Serviço Social e Investigação Social, Universidade Lusíada, Lisbon, Portugal

**Keywords:** Moreno, psychodrama, techniques, group therapy, review

## Abstract

The original theory of psychodrama proposed by Moreno in 1921 has been adjusted and re-interpreted by several authors over the last three decades. This resulted in the proliferation of techniques whose definitions and contexts of application are unclear and poorly documented in the literature. The purpose of this review was three-fold: (1) to identify the psychodramatic techniques currently used for research and clinical purposes, (2) to extract and create a list of core techniques which are consensually used by psychodramatists, and which reflect the main principles of the Morenian theory of psychodrama, and (3) to propose an operationalised definition of the core psychodramatic techniques identified. To achieve this, a systematic review was conducted, according to the PRISMA guidelines (Moher et al., 2009). The search was conducted between June and September of 2012 in the main electronic databases (e.g., PubMed, Embase, PsychINFO) and using the following keywords: “psychodrama,” “group psychotherapy,” “experiential psychotherapy,” “Moreno,” “intervention,” and “techniques.” Fifty-six techniques were extracted from the 21 papers selected for review. Of these, a preliminary list of 30 techniques was selected, which was reduced to a total of 11 core techniques: soliloquy, double, mirror, role reversal, resistance interpolation, sculpture, social atom, intermediate objects, games, sociometry, role training. The credibility of this final core list was first checked with an expert in Morenian psychodrama, and later discussed with a network of 22 European psychodramatists to ensure full consensus. Overall, this review provides a contemporary framework for psychodramatists that reconciles the current approaches to psychodrama with the core techniques proposed by Moreno, and updates the definitions of these techniques, by merging the interpretations of different experts in the field. To have a list of core techniques which is consensually accepted from an international point of view is paramount not only for future research, but also for training purposes. The implications of this review for clinical practice are also discussed.

## Introduction

Created by Moreno in 1921 (Moreno, 1946/1993), psychodrama is a therapeutic model widely used in Europe in private and public health settings, including hospitals (e.g., Vieira et al., 1993; Kipper, 1997; Sousa, 2012) and mental health services (e.g., Kirk and Dutton, 2006), in the treatment of various pathologies such as schizophrenia (e.g., Parrish, 1959; Sousa, 2012) and substance abuse (e.g., Crawford, 1989; Couto, 2004; Pinheiro, 2004). Because of this, psychodrama has been accredited by the European Association of Psychotherapies (EAP) and is also recommended by several European governments as a good health practice, such as in Austria and Hungary. In the last decades, the growing popularity of psychodrama has led to the proliferation of over 60 psychodrama training and accreditation schools over 26 European countries. Most of these schools are overseen by the European Federation of Psychodrama Training Organizations (FEPTO, http://www.fepto.com). FEPTO aims to develop training, create ethical standards and promote scientific knowledge sharing across trainers and schools. However, it is unclear which psychodrama techniques are currently being used and taught, and whether this proliferation of schools has fragmented the original theory proposed by Moreno, to which the present review will contribute.

Psychodrama is a group format of psychotherapy with deep roots in theater, psychology and sociology. Although preferably performed in a group format, it focuses on the particularities of the individual as the intersection of various relational roles, (e.g., being a son and a spouse) and roles related to difficulties and potentialities (e.g., fears, like fear of flying; or doubts, how the next job interview will be). For this reason, it is said to be an individual therapy in a group format, centered on the protagonist, and the action may take place around the various roles that s/he assumes throughout life (Blatner, 1996). A psychodramatic session comprises of three contexts: social, group and dramatic (Gonçalves et al., 1988; Rojas-Bermúdez, 1997); five instruments: protagonist, stage, auxiliary-ego, director, audience (Moreno, 1946/1993; Gonçalves et al., 1988; Holmes, 1992; Pio de Abreu, 1992; Rojas-Bermúdez, 1997); and three distinct phases: warm-up, action, and sharing (Moreno and Moreno, 1975/2012; Gonçalves et al., 1988; Holmes, 1992; Kipper, 1997). The majority of the techniques found in the literature, such as role reversal, soliloquy, or double mirroring, are used to assist the protagonist in the dramatization of the conflict that needs to be solved. Others, however, can be used both as a warm-up for the action phase and emergence of the protagonist, as well as to work out a common topic for the whole group and to constitute the stage of the drama itself. This is the case of dramatic games and sociometry.

From the early 1980s, several authors suggested deviations from the original psychodramatic theory. These suggestions were, on the one hand, attempts to demonstrate the integration of Moreno's methods and ideas with other theories (Holmes, 1992; Blatner, 1996) and, on the other hand, to conceive new theoretical bases for the method (Kipper, 1982, 1997; Rojas-Bermúdez, 1997). With this separation from the original formulations, a deviation from the traditional dynamic of the session also occurred (warm-up, action, sharing), with the application of specific psychodramatic techniques as independent interventions within the more traditional, verbal psychotherapy (Kipper, 1997).

The dissemination of psychodrama across the different countries of Europe and America and the absence of clear definitions has resulted in a diversity of applications of the techniques and concepts introduced by Moreno within psychodrama itself. Therefore, the practice of psychodrama has evolved in an isolated and distinct way across various countries and schools, and there are no common definitions of some of its components, namely the techniques. Also, when it comes to the operationalization of the model, the techniques seem to be its component that meets less consensus. In short, answering the question “what does define Moreno's psychodrama” has become a challenge. Hence, in a time when it is considered important to study psychodrama and to stimulate research, a fundamental and key step is to operationalize the model. It is essential to realize, within Morenian Psychodrama (MP), what is being done and how, which techniques are being used and which techniques constitute the basis of the theory.

The present review aims to contribute to the understanding of how MP has evolved and the way it has been practiced since the launching of its theoretical roots. We will achieve this through the systematization of core techniques used at an international level. More specifically, our review aims:

1. To identify MP techniques existing in the international literature; and
2. To identify and describe the techniques that gather consensus across the community of researchers and practitioners of MP.

## Methods

### Search strategy

This review followed, the procedure suggested by the guidelines “Preferred Reporting Items for Systematic Reviews and Meta-Analyzes (PRISMA)” (Moher et al., 2009). The search for literature sources occurred between June-September 2012 and included the databases Scopus, PsycINFO, PsycARTICLES, B-on, Psychology and Behavioral Sciences Collection, SciELO and Bibliography of Psychodrama Database^1^ (http://www.pdbib.org). The keywords used in the search were “psychodrama,” “group psychotherapy,” “experiential psychotherapy,” “Moreno,” “intervention,” and “techniques.” In the pdbib database, only the terms “intervention” and “techniques” were used as keywords, since this bibliographic source was specific for psychodrama. Additionally, internet search engines were also searched (such as Google), as well as gray literature (e.g., books, articles, master's theses, and doctoral theses in psychodrama). Finally, national and international psychodrama experts (psychologists and psychiatrists) were contacted to identify relevant studies/texts/books for review. This contact was made in person and via e-mail.

### Eligibility and selection criteria

**Figure 1:**
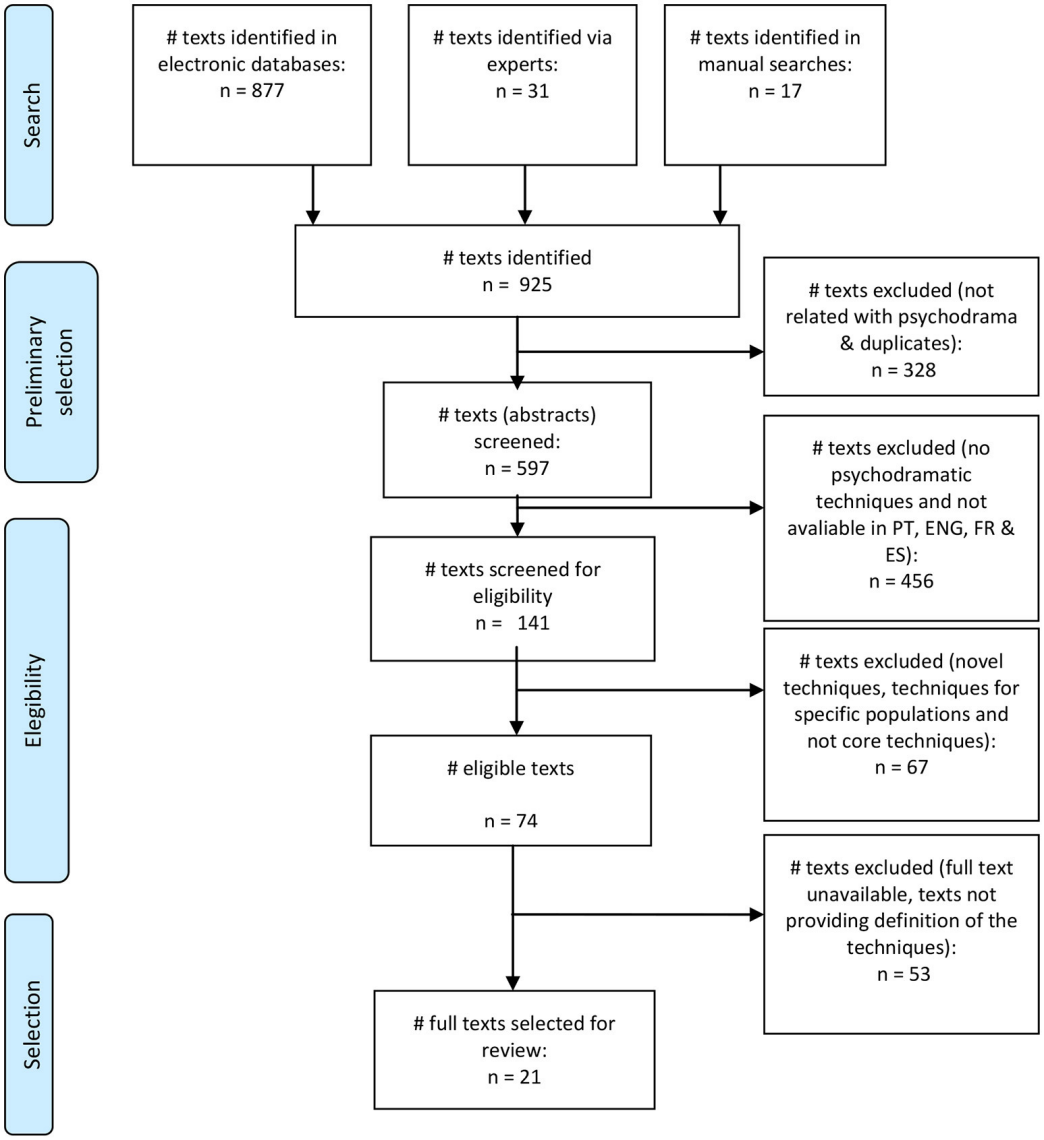
Selected texts. Adapted from Moher et al. (2009).

After the systematic search, the texts were selected according to the following inclusion criteria: (1) texts that described or indicated psychodramatic techniques; and (2) available in Portuguese, French, English, and Spanish. These criteria were chosen because the goal was to identify all existing techniques, even if their definition was not entirely clear (see section on quality assessment of texts selected for review). As for languages, the research team included those in which they had fluency to understand and review. The following exclusion criteria were adopted: (1) information from websites without peer review; (2) texts of unknown origin (e.g., without author, list of references or bibliographical citations), and (3) texts referring to new techniques or techniques applied to specific populations only. These exclusion criteria aimed to ensure that all selected texts came from reliable sources, included reliable contents from an academic and scientific point of view and referred to the original MP model. Finally, the preliminary list of selected studies was verified by an expert in psychodrama. All disagreements were discussed until consensus was reached on the final list of texts to be included for review (see diagram in Figure 1).

### Selection of the final list of MP techniques

MP techniques were identified and extracted from the texts selected for review. The following techniques were excluded from the preliminary list: techniques mentioned only once in the literature; specific techniques for certain pathologies; and techniques that were directly related to therapeutic modalities^2^. This list was then discussed with a psychodrama expert, until a final list of core techniques, which were consensual and could be traced back to the model proposed by Moreno, was prepared.

The final step involved the validation of the final list of MP techniques by international experts in psychodrama. For this, the relevance of the techniques and their definition were discussed by experts in a bi-annual meeting of the FEPTO Research Committee in October 2012. In this meeting, all techniques were discussed by 22 members of FEPTO, representing a total of 11 countries^3^, until a consensus was reached on the completeness of the list and the operational definition of each technique.

### Quality check

The quality of the papers selected for review was evaluated according to two criteria: the reliability of the source, considering peer recognition in the scientific and clinical community; and the clarity of the definition of the techniques provided in each paper.

To evaluate the reliability of the source, a point-based evaluation system (see criteria in Table 1) was used to value peer-reviewed periodicals (1 point), in contrast to publication not reviewed by peers/status unknown (0 points). Books and gray literature (e.g., theses) received 1 point if written by recognized specialists in the field (i.e., pioneer in psychodrama, trainer in Psychodrama, or affiliation to training schools or training centers) or certified psychodramatists; and received 0 points if the author was not a recognized specialist (unknown) or whose training was not accredited.

**Table 1:** Checklist for assessing sources quality and techniques definition.

**Criteria**		**Condition**	**Score**
Authorship/Peer Recognition	Articles	Peer-reviewed periodical publication	1
		Publication not reviewed by peers / status unknown	0
	Books or gray literature (e.g., theses)	Specialists recognized in the field or certified psychodramatists	1
		Unknown author, unknown training or non-accredited training.	0
Clarity of techniques definitions found in various sources		Absolutely clear definitions	1
		Incomplete or unclear definitions	0

All sources were classified according to these parameters. For quality check purposes, a second independent judge (clinical psychologist with accredited training in psychodrama) classified a random sub-sample of 50% of the sources, resulting in a 100% agreement.

To evaluate the quality of the definitions, we focused on the clarity of the operational domains, objectives and advantages of each technique (see Table 1). One point was assigned when the definition was clear and 0 points when the definition was considered incomplete or unclear. A second independent judge (clinical psychologist with accredited training in psychodrama) evaluated 50% of the definitions and the agreement between the two judges was calculated through Cohen's Kappa, with the following results: role reversal (*k* = 0.87), mirror (*k* = 0.63), resistance interpolation (*k* = 0.70), role training (*k* = 0.68), dramatic games (*k* = 1) and amplification (*k* = 0.40)^4^.

## Results

A total of 925 texts were found in the systematic search, of which 904 were excluded. Out of this search, 21 texts were initially selected for review, which comprised of 15 books and six articles. In terms of quality, all the books scored 1 point. Of the six articles, only one was not a peer-reviewed publication, receiving 0 points and thus being excluded, whilst the remaining five received a score of 1 point. This resulted in a final list of 20 texts to be used for the extraction of MP techniques.

### Core MP techniques

**Figure 2:**
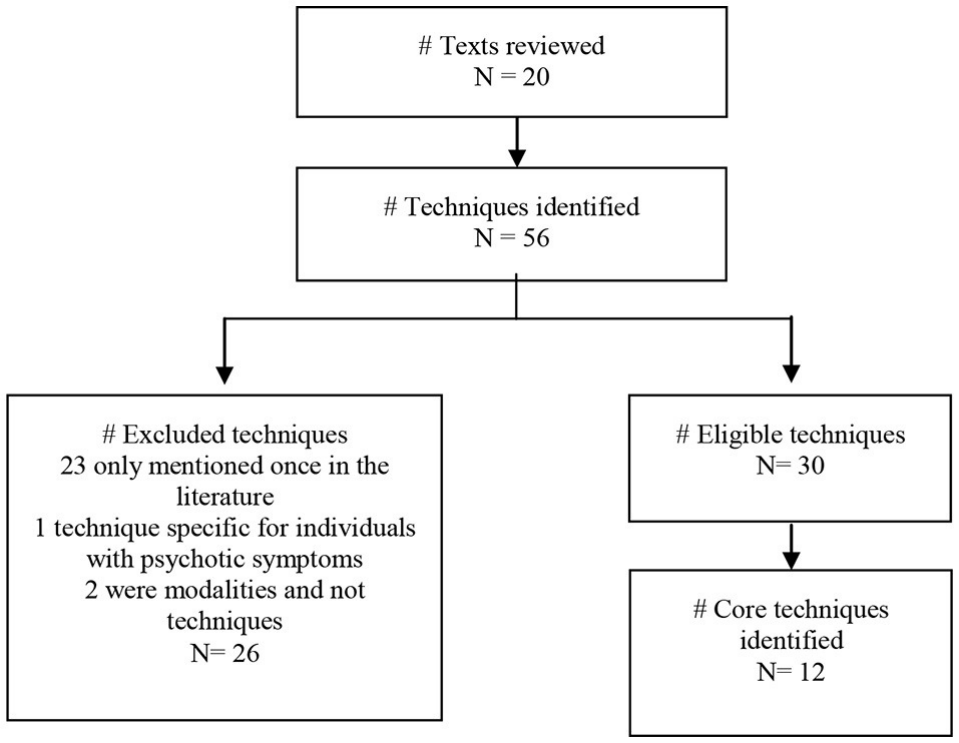
Selection process of the MP techniques.

Fifty-six techniques were initially extracted from the 20 texts. Of these, 30 were considered eligible for selection, among which 12 MP core techniques were identified. Figure 2 and Table 2 provide further details about the selection process and Annex 1 presents a list of the total 56 techniques that were identified in this search.

**Table 2:** List of core techniques validated by FEPTO-RC.

	**List of techniques initially proposed**	**Techniques selected by FEPTO-RC**	**Sources used for the definition of the techniques (quality = 1 point)**
Core techniques	Soliloquy Double Mirror Role Reversal Resistance Interpolation	Soliloquy The director asks the protagonist to think “out loud” and express his/her feelings, thoughts or intentions	Pio de Abreu, 1992; Rojas-Bermúdez, 1997; Santos, 1998; Cukier, 2002
		Double The auxiliary ego plays the role, or an aspect of protagonists' role, by standing to the side or behind him/her; expressing the protagonist's unspoken thoughts and feelings.	Gonçalves et al., 1988; Holmes, 1992; Pio de Abreu, 1992; Blatner, 1996; Rojas-Bermúdez, 1997; Gonçalves, 1998; Cukier, 2002; López, 2005
		Mirror The protagonist watches, as if in a mirror, the auxiliary ego playing his or her role, reproducing it by mirroring his/her postures, gestures and words as they appeared in the dramatization.	Gonçalves et al., 1988; Pio de Abreu, 1992; Rojas-Bermúdez, 1997; Gonçalves, 1998; Cukier, 2002; López, 2005
		Role reversal A dramatization in which the protagonist reverses with other roles, so that the protagonist places him/herself in the other's shoes	Blatner and Blatner, 1988; Holmes, 1992; Pio de Abreu, 1992; Kellerman, 1994; Rojas-Bermúdez, 1997; Cukier, 2002; López, 2005
Secondary techniques	Sculpture Social atom Intermediate objects Games Sociometry Role play Symbolic representation	Resistance Interpolation The director asks the auxiliary ego to act in a completely different way to which the protagonist would expect (e.g.: an authoritarian figure may become humble and compliant).	Gonçalves et al., 1988; Pio de Abreu, 1992; Rojas-Bermúdez, 1997; Calvente, 1998; López, 2005
		Sculpture The director asks the protagonist to arrange group members in a symbolic representation of the way he/she perceives an aspect of his/her life or self.	Pio de Abreu, 1992; Hug, 1997; Rojas-Bermúdez, 1997; López, 2005; Moyano, 2012; Rojas-Bermúdez and Moyano, 2012
		Social atom Representation or configuration of all the meaningful relationships in protagonists' life. It can be represented in diagrams or graphic terms, or about individuals or issues, in past or present terms, intensity and/or distance.	Gonçalves et al., 1988; Pio de Abreu, 1992; Cukier, 2002
		Intermediate Objects The director introduces the use of objects in the session to facilitate communication with the protagonist (e.g. a doll, puppet, stone, fabrics, etc.).	Rojas-Bermúdez, 1997, 2012
		Games A game with specific objectives and specific rules	Pio de Abreu, 1992; Rojas-Bermúdez, 1997; Monteiro, 1998
		Sociometry Measure interpersonal relationships, how group members position themselves in relation to each other, in response to given criteria.	Blatner and Blatner, 1988; Gonçalves et al., 1988; Fox, 2002
		Role training To practice a role, to simulate a situation, to try different answers, alternatives or behaviors.	Boies, 1972; Blatner and Blatner, 1988; Pio de Abreu, 1992; Soeiro, 1995; Rojas-Bermúdez, 1997; Kaufman, 1998; Cukier, 2002
Other techniques or actions		Symbolic representation Amplification Concretization Empty chair	–

As Table 2 shows, some changes were made to the initial proposal of MP core techniques, following the feedback of FEPTO-RC experts. These changes, to which we refer next, were due to the consensual meeting of the differences between the different schools.

1. Resistance Interpolation was presented as one of the main techniques of psychodrama. Many of the schools represented in the meeting were not aware of this technique and, after discussion, this consensually classified as secondary.
2. Role-play raised the theoretical issues mentioned below, and was later designated as role training;
3. Symbolic representation, also proposed as a secondary technique, was rarely used by many of the schools and agreement was not reached about its theoretical definition. Even though used to represent difficult situations on stage, such as sexual intercourse, this was considered by some experts as a psychodramatic principle and not as a technique. This discussion led to the creation of a new category “other techniques,” where this was included;
4. Amplification, concretization, symbolic representation and empty chair were added to the category “other techniques.”

## Discussion

The main objective of this study was to identify contemporary MP core techniques, as used in real clinical practice and to propose an updated definition to those MP techniques, which were consensually agreed by a group of international experts and certified trainers in this field.

### Soliloquy

Soliloquy is a technique brought by Moreno directly from classical theater where it had artistic aims (Moreno, 1946/1993; Santos, 1998). It was described approximately in half of the revised texts (10 out of 20), and was one of the most consensual techniques in terms of its operability.

For Moreno (1946/1993), the “purpose is to be cathartic” (p. 245), and its “end is the knowledge of oneself” (Moreno, quoted in Cukier, 2002, p. 307). The intention is for the protagonist to externalize the hidden feelings and thoughts (Rojas-Bermúdez, 1997; Moreno, quoted in Cukier, 2002), “to reveal deeper levels of the interpersonal world” (Moreno, quoted in Cukier, 2002, p. 306). “It allows correcting any misrepresentations of the scene, being valuable for the adaptation of the auxiliary egos and orientation of the director (…) If the dramatization ends in this way, one can obtain the 'insight' of the protagonist” (Pio de Abreu, 1992, p. 30). The protagonist has the opportunity to change and integrate into action, what s/he expressed in soliloquy (Rojas-Bermúdez, 1997).

When the protagonist holds his/her action or becomes ambivalent, the director asks him/her to “think out loud” (Rojas-Bermúdez, 1997), outside the dramatization dialogue, expressing what s/he thinks and feels in the here-and-now (Pio de Abreu, 1992; Rojas-Bermúdez, 1997; Santos, 1998). Soliloquy can also be performed as the protagonist walks the stage (Santos, 1998).

### Double

The double technique was referred to in 14 of the 20 texts, and is considered by Moreno (quoted in Cukier, 2002, p.310), “as old as civilization. We find it in the great religions. I have often thought that God must have created us twice, one for us, to live in this world, and another for ourselves.” This technique is used to (a) assist the protagonist in the expression of thoughts and feelings that, for some reason, s/he does not perceive or avoids expressing both verbally and bodily (Blatner and Blatner, 1988; Rojas-Bermúdez, 1997); (b) support the protagonist to enter the dramatization more fully and deeply (Blatner and Blatner, 1988); (c) test the director's interpretation of the protagonist's inner messages by means of an auxiliary ego (Pio de Abreu, 1992; Gonçalves, 1998); and (d) be a vehicle to provide more effective suggestions and interpretations to the protagonist (Blatner and Blatner, 1988). By identifying with the double, the protagonist may gain insight (Gonçalves et al., 1988). The double can also constitute a good warm-up for the auxiliary ego (Gonçalves, 1998).

While the protagonist represents his/her own role, the auxiliary ego stands beside or behind him/her, adopts his/her body and emotional expression, and slowly adds the emotions, fears, motives, or hidden intentions that the protagonist is not explicit about (Gonçalves et al., 1988; Holmes, 1992; Pio de Abreu, 1992; Rojas-Bermúdez, 1997; López, 2005). It is therefore a procedure that requires corporal flexibility and telic sensitivity on the part of the therapist and auxiliary ego (Gonçalves, 1998).

One can make subsequent or simultaneous doubles. This is useful when one wants to know the opinions of the group members regarding, for example, a dramatized scene (Rojas-Bermúdez, 1997). Each element of the audience should, in turn, place a hand on the shoulder of the protagonist and while doubling, will say what s/he feels from the role of the protagonist. This way of applying the double allows a minimization of the negative impact of feeling imitated (Rojas-Bermúdez, 1997; Gonçalves, 1998).

### Mirror

Mirror was found in approximately half of the revised texts (11 out of 20), and although it can be applied in various ways, there were no significant disagreements regarding its definition. The purpose of this technique is to promote the awareness of the protagonist and his/her behavior in different situations (López, 2005). It is used when the protagonist does not perceive his/her behavior, and the image s/he transmits to others differs substantially from the image s/he has of him/her self (internal and external image) (Pio de Abreu, 1992; Rojas-Bermúdez, 1997). As Moreno conceived its aim is to transform the protagonist into a spectator of him/herself.

It can be applied in a variety of ways: in the dramatization, the auxiliary ego imitates the protagonist, standing in front of him/her, saying and doing what s/he does (Gonçalves et al., 1988; Rojas-Bermúdez, 1997); once the dramatization is finished, the auxiliary ego reproduces what the protagonist dramatized while s/he observes from the audience (Gonçalves et al., 1988; Rojas-Bermúdez, 1997; Gonçalves, 1998; Moreno, quoted in Cukier, 2002; López, 2005). An alternative option can be used, the “technological mirror,” which may rely on photography, cinema, video and audio recordings to achieve similar results (Rojas-Bermúdez, 1997).

This technique can be potentially uncomfortable and provocative for the protagonist. As such, it is recommended that a professional auxiliary ego is used to avoid the risk of the protagonist feeling ridiculed (Pio de Abreu, 1992; Rojas-Bermúdez, 1997).

### Role reversal

Role reversal is one of the foundations of Moreno's theory (Rojas-Bermúdez, 1997) and was the most common technique in the literature, present in 15 out of 20 texts.

This technique allows the protagonist to obtain a more accurate perception of the individuality of the complementary role (López, 2005), as well as the possibility of perceiving the other's view about him/herself (Kellerman, 1994), and about the world (Holmes, 1992). It also allows a characterization of the characters so that the auxiliary ego learns the role (verbal and non-verbal component) that has been assigned to him/her. This warms up for the action so that the represented scene is as close as possible to the protagonist's experience (Blatner and Blatner, 1988; Pio de Abreu, 1992; Rojas-Bermúdez, 1997).

In a dramatization, the protagonist is invited by the director to reverse with the other with whom s/he interacts, namely, the complementary role (hereby referred to as auxiliary ego). This auxiliary ego can be an element of the therapeutic team or an element of the audience. With role reversal, the protagonist places him/herself psychologically in the place of this other person (Pio de Abreu, 1992).

### Resistance interpolation

Found only in one third of the texts, and little known among the elements of the FEPTO-RC, this technique has also been seen as a concept.

The interpolation of resistances may be used to test the capacity of the protagonist to face a situation (López, 2005): when it is used unexpectedly, it will test the spontaneity of his/her response, while providing an opportunity to train his/her flexibility and discover new possibilities in face of an unfavorable situation (Pio de Abreu, 1992). It can also be used to corroborate a diagnostic hypothesis: if the results are not obtained, the hypothesis should be abandoned (López, 2005).

As a technique, it consists on the modification, by the director, of the scene presented by the protagonist. S/he presents his/her scene according to his/her point of view, based on an argument and certain expectations about its outcome. The director introduces modifications (e.g., modifies the characteristics of the dramatic context and/or the complementary roles) through indications to the auxiliary ego: introducing unforeseen factors that lead the protagonist to act spontaneously, revealing forms of behavior and personality (Pio de Abreu, 1992; Rojas-Bermúdez, 1997; López, 2005). “An authoritarian character can become humble and submissive, an attentive individual can become deaf or distracted, a docile relative can become irascible” (Pio de Abreu, 1992, p. 31).

### Sculpture

Referred to in eight of the 20 texts, the origin of this technique was not clear. The school of Rojas-Bermúdez speaks of psychodramatic images and distinguishes them from the concept of Moreno's therapeutic images (quoted in Cukier, 2002). For this clarification, we considered important to compare three concepts: sculptures, psychodramatic images and therapeutic images. Moreno refers to therapeutic imaging as a “method that can be used with advantage (…). The method of image activation is only a resource to assist the musician or student in the process of learning to be spontaneous” (Cukier, 2002, p.150), but it is not clear in his definition as a technique. Rojas-Bermúdez and Moyano (2012) state that, although Moreno used the term, he referred to it as a mental image.

Rojas-Bermúdez and Moyano (personal communication, February 10, 2012) claim to prefer the term “images” to “sculptures” because they understand that the latter was taken from other therapeutic approaches. Blatner (1996, 1997) argues that sculpture is traditionally seen as a family therapy technique and is an adaptation by Virginia Satir of the psychodramatic technique action sociogram (Blatner, 1997). When consulted directly by e-mail, Zerka Moreno (personal communication, February 20, 2012) clarified that Moreno would have suggested sculpture to one of his students as a family organization. Rojas-Bermúdez and Moyano (2012) also claim to have been introduced to this technique in the scope of family therapy. Some psychodramatists following a systemic perspective have, since 1990, been incorporating this technique into their work, considering sculptures as an expression of the binding structure of a system.

The objective of the sculpture technique is the observation by the protagonist, the director and the group, of the organization within his/her sculpture figure, the connections between its elements and the exploration of their meanings. This technique is used to deepen the knowledge of a certain material. When constructed by the protagonist him/herself, s/he “drags” his/her characteristics and, therefore, allows a quick access to his/her contents (Rojas-Bermúdez, 2012).

The protagonist is asked to construct a figure (with people or objects) that represents the material brought by him/her. The protagonist must choose an auxiliary ego to represent himself/herself (Rojas-Bermúdez, 1997). The starting point for its construction can be directly the mental image (for example, a dream, a fantasy, a memory), or a mental image corresponding to a word (e.g., “duel”), or a phrase (e.g., “I feel sunk”); it can be a construct elaborated to convey a state of mind (e.g., sadness) or a physiological process (e.g., hunger) (Rojas-Bermúdez, 2012). From this first image, other images may be requested in a temporal line (before, after), other spaces (in parallel, in another place), contrasting values (better, worse, pleasant, unpleasant), reference points for improvisations that integrate several images (to invent a story, to tell a story), among others (Rojas-Bermúdez, 1997).

Usually, sculptures tend to be realistic and constructed with elements of the group, but they can also be symbolic and accomplished with both people and objects (Pio de Abreu, 1992; Rojas-Bermúdez, 1997).

### Social atom

The social atom, referred to in 10 of the revised sources, is described by Moreno (1946/1993) as “the nucleus of all individuals with whom a person is emotionally related or at the same time related to it. It is the minimal core of an emotionally accentuated interpersonal pattern in the social universe. The social atom reaches as far as tele itself reaches other people. Therefore, it is also called the tele-range of an individual” (p.289). It is a technique of presentation of the protagonist through which s/he presents the significant others of his/her life (Gonçalves et al., 1988; Pio de Abreu, 1992), often used in initial interviews and diagnoses (Gonçalves et al., 1988). The social atom provides an overview of the protagonist's interpersonal structure, revealing conflicts with significant people and providing themes for dramatization.

Family members and significant others are arranged in the scenario, represented by auxiliary egos and also objects. Distances, positions and postures are important elements. The protagonist makes role inversions with each of the people represented (Gonçalves et al., 1988; Pio de Abreu, 1992). The reversal of roles with significant others reveals common interactions and the protagonist's understanding of them (Pio de Abreu, 1992).

### Intermediate objects

Described in six of the 20 texts, in all of them the concept is recognized as being of Rojas-Bermúdez (Pio de Abreu, 1992; Blatner, 1997; Hug, 1997; Rojas-Bermúdez, 1997; López, 2005; Rojas-Bermúdez et al., 2012). Although objects have always been used, Rojas-Bermúdez owes the concept and theoretical framework. It is important to mention that although this was not a concept of Moreno, the use of different objects was suggested and is part of all Psychodrama schools, and hence this was consensually considered as one of the MP most important techniques.

Objects such as props, fabrics, puppets, cloth dolls and masks have been recognized as catalysts of important non-verbal reactions and at the same time allow a greater distance from the emotionally charged situation (Blatner, 1997). In its simplest form, it is an articulated doll that, through the voice of the director, “talks” with the protagonist (Pio de Abreu, 1992).

According to Rojas-Bermúdez (1997), it allows the reestablishment of interrupted communication with the patient, replacing the direct therapist-patient relationship with object-patient, in order to facilitate the focus of attention and decrease alarm states.

When the patient does not respond to verbal communication, the professional auxiliary ego addresses the patient through the object (puppet, mask, hood, tunic); and based on the patient's reaction, the auxiliary ego can continue to use the object, or choose another object, or give the patient a similar object to interact with. When face-to-face communication is achieved, the object is eliminated (Rojas-Bermúdez, 1997).

### Games

Dramatic games were referred to in about a quarter of the revised references. The game must go through the same stages of the psychodrama session: warm-up, action and sharing (Monteiro, 1998).

There is a wide variety of games ranging from improvisation and character play to collective creation (Rojas-Bermúdez, 1997). The main objective is to provide an opportunity to freely express the inner world and externalize a fantasy through the representation of a role, or bodily activity (Monteiro, 1998). In the warm-up phase, games aim to raise therapeutic material to decide the theme of the session and/or the protagonist (Pio de Abreu, 1992; Soeiro, 1995; Monteiro, 1998). Particularly useful to increase group cohesion, it strengthens the trust among the members, creates a relaxed atmosphere, resolves intra-group tensions, and changes the focus of a group that is constantly around recurring issues (Pio de Abreu, 1992). Although they are play activities, they reflect personal aspects that can help the director to move from the game to the reality (Rojas-Bermúdez, 1997).

### Sociometry

Referred in eight of the 20 texts, one of the challenges presented by sociometry concerns its conceptual diversity, which probably comes from the importance and comprehensiveness that it has assumed over time.

It has been considered as a scientific method to objectively determine the basic structure of human societies (Fox, 2002), as well as a method to measure interpersonal relationships (Blatner and Blatner, 1988). Its purpose is to help the elements of a group to provide mutual feedback on various issues (Blatner, 1997). As a technique, it is used to measure interpersonal relationships (Blatner and Blatner, 1988; Gonçalves et al., 1988) regarding the criteria of interest to the researcher and how to warm up for group interactions (Blatner and Blatner, 1988). It makes isolated people to stand out, making visible the pattern of the social universe (Blatner and Blatner, 1988; Fox, 2002).

Sociometric data can be obtained in writing: each element registers their choice of other members of the group according to the criteria presented by the director. The choices are all placed in a diagram or table and then the results are shared with the group (Blatner and Blatner, 1988). They can also be obtained in action: by placing a hand on the shoulder of the selected person. This alternative is termed “sociometric action” because interpersonal choices are displayed in action, and used when immediate feedback is needed (Fox, 2002). After making the choices, there is room for confrontation and clarification among the participants (Gonçalves et al., 1988).

### Role training

Here it is important to note that all the definitions (12 out of 20) were reviewed, both for role-playing and role-training, so that better theoretical support could be made.

Role training aims to create situations for the development and training of a certain role in conditions very close to the real situation yet in a protected way (Blatner and Blatner, 1988; Soeiro, 1995). It can be used as well as a diagnostic method (Moreno, cited in Cukier, 2002).

Essentially, “it consists of representing a role whose performance is feared, for example, that of a student during a next examination, or a role usually played poorly, such as the boss who does not know how to give orders” (Pio de Abreu, 1992, p. 37) and can be operationalized in two ways: the person is asked to play a role that is not normally theirs (Boies, 1972; Cukier, 2002), or the person is asked to play his/her own part, but not in the setting in which it is normally played (Boies, 1972).

## Conclusion

Almost 100 years after its foundation MP still lacks theoretical and technical coherence within the international clinical community. We believe this work is a contribution to take the first step in that direction by finding the 11 consensual core techniques that are used mainly in the action phase of the traditional psychodrama session in contemporary Morenian Psychodrama. Psychotherapeutic and integrative models have been making extensive use of MP techniques. Techniques such as role reversal, sculpture, empty chair, and others can be used during a session, without the need for a group or auxiliary egos (Blatner, 2007). This fact points us to the importance and clinical relevance of the method. However, when used outside of its theoretical and philosophical frame of reference, these techniques may become distorted (Bustos, 1999). In fact, many of Moreno's original techniques have been appropriated by other theoretical models, which led many of their users to be unaware of their origin (Blatner and Blatner, 1988; Bustos, 1999). For instance, this is the case of sculpture in family therapy and the use of the auxiliary chair by Fritz Perls in Gestalt Therapy, which was later modified for the “two-chair” technique in the cognitive approach (Blatner and Blatner, 1988; Blatner, 1997). Hence, it is important that individual, verbally-based psychotherapies acknowledge the basic principles of MP techniques, such as those highlighted by this review. Respecting these basic principles will, therefore, prevent a potential disconnection between MP techniques and their theoretical roots, allowing them to evolve and become fully integrated with other therapeutic models.

This review is not without limitations. The first is the exclusion of texts whose languages were not fluently spoken by our research team, leading to many articles e.g., in German and Italian to be excluded. To overcome this limitation, the results were validated and discussed with representatives of several countries included in FEPTO, to widen the scope of this review. As for peer-reviewed papers included in the review, these tended not to include enough information to define the techniques. However, there is a growing tendency for studies on the effectiveness of techniques individually (Kipper and Ritchie, 2003). This was also expected considering that definitions of techniques are usually published for didactic and training purposes and less frequently in empirical articles.

## Author contributions

AC was responsible for conducting the review, analyzing the data and writing the manuscript. CS and GM supervised the review and contributed for the manuscript. PA collaborated with the review and contributed for the manuscript. All authors approved the final version of this manuscript. This review was conducted as part of the AC research project toward a Ph.D. degree, of which CS and GM were the supervisors.

### Conflict of interest statement

The authors declare that the research was conducted in the absence of any commercial or financial relationships that could be construed as a potential conflict of interest.

## Footnotes

^1^Compiled and updated by James M. Sacks until 2009, followed by Michael Wieser. Contributions by Valerie Greer, Jeanine Gendron, and Marie-Therese Bilaniuk.

^2^An example of this is the “Improvisation Theater,” a theater modality founded by Moreno in 1921.

^3^Germany, Austria, Bulgaria, Finland, Hungary, Israel, Italy, Portugal, United Kingdom, Serbia and Switzerland. This discussion was also promoted to validate Helpful Aspects of Morenian Psychodrama Content Analysis System manual (Cruz, 2014; Cruz et al., 2016).

^4^Despite being considered statistically low, this value of *k* = 0.40 was considered acceptable for this study since it results from the agreement between 3 ratings only, i.e., a case in which the judges only had to evaluate the quality of only 3 definitions, being that, in this case, the discrepancy between only one value (as has been seen in practice) was sufficient to lower the value from 1 to 0.4.

## Annex 1

### Initial list of 56 MP techniques identified in the review

Live wheel; gallery mirror; intermediate scenes; therapeutic objectivators; aesthetic communication techniques; autodrama; replay; monodrama; aside; multiple ego; crib scene; structured negotiation; breaking in; spectrogram; nonverbally coming together; shared secrets; directed fantasy; chorus; maximization; substitute role; projection; symbolic distance; closure; auxiliary world; improvisational theater; living newspaper; role reversal; soliloquy; mirror; double; sculpture; resistance interpolation; social atom; intermediate and intraintermediate objects; dramatic games; sociometry; role-playing; symbolic representation; amplification; concretization; empty chair; surplus reality; self presentation; behind back; puppets and masks; psychodance; body techniques; psychomusic; hypnodrama; magic shop; onirodrama; spontaneous improvisation; videopsychodrama; future projection; spontaneity test.

### List of MP techniques considered as eligible for selection

Role Reversal; Soliloquy; Mirror; Double; Resistance Interpolation; Sculpture; Social atom; Intermediate and Intraintermediate Objects; Dramatic Games; Sociometry; Role training; symbolic representation; amplification; concretization; empty chair; surplus reality; self presentation; behind back; puppets and masks; psychodance; body techniques; psychomusic; hypnodrama; magic shop; onirodrama; spontaneous improvisation; videopsychodrama; future projection; spontaneity test.
